# Non-disulfide-Bridge Peptide 5.5 from the Scorpion *Hadrurus gertschi* Inhibits the Growth of *Mycobacterium abscessus* subsp. *massiliense*

**DOI:** 10.3389/fmicb.2017.00273

**Published:** 2017-02-22

**Authors:** Monalisa M. Trentini, Rogério C. das Neves, Bruno de Paula Oliveira Santos, Roosevelt A. DaSilva, Adolfo C. Barros de Souza, Márcia R. Mortari, Elisabeth F. Schwartz, André Kipnis, Ana P. Junqueira-Kipnis

**Affiliations:** ^1^Laboratory of Immunopathology of Infectious Disease, Tropical Institute of Pathology and Public Health, Department of Microbiology, Immunology, Parasitology and Pathology, Federal University of GoiásGoiânia, Brazil; ^2^Collaborative Center of Biosystems, Regional Jataí, Federal University of GoiásGoiânia, Brazil; ^3^Laboratory of Neuropharmacology, Department of Physiological Sciences, Institute of Biological Sciences, University of BrasíliaBrasília, Brazil

**Keywords:** AMP, antimycobacterial agents, infection, non-disulfide bridged peptide, rapid-growing mycobacteria, scorpion venom

## Abstract

Multi-drug resistant microorganisms have been a growing concern during the last decades due to their contribution in mortality rates worldwide. Antimicrobial peptides (AMPs) are broad spectrum antimicrobial agents that display potent microbicidal activity against a wide range of microorganisms. AMPs generally have a rapid mode of action that reduces the risk of resistance developing among pathogens. In this study, an AMP derived from scorpion venom, NDBP-5.5, was evaluated against *Mycobacterium abscessus* subsp. *massiliense*, a rapidly growing and emerging pathogen associated with healthcare infections. The minimal bactericidal concentration of NDBP-5.5, AMP quantity necessary to stop bacteria visible growth, against *M. abscessus* subsp. *massiliense* was 200 μM, a concentration that did not induce hemolysis of human red blood cells. The therapeutic index was 3.05 indicating a drug with low toxicity and therefore good clinical potential. Treatment of infected macrophages with NDBP-5.5 or clarithromycin presented similar results, reducing the bacterial load. *M. abscessus* subsp. *massiliense*-infected animals showed a decrease in the bacterial load of up to 70% when treated with NDBP-5.5. These results revealed the effective microbicidal activity of NDBP-5.5 against *Mycobacterium*, indicating its potential as an antimycobacterial agent.

## Introduction

Over the last decade, a significant increase in the number of studies investigating the potential use of antimicrobial peptides (AMPs) to treat diseases has been observed (Gordon et al., 2005). AMPs are positively charged (+1 to +9) peptides comprising 12–100 amino acids, produced by the cells of insects, plants, amphibians, and mammals, that exert microbicidal function (Zeng et al., 2005; Ortiz et al., 2015). Although the mechanisms involved in the microbicidal function of AMPs remain to be fully elucidated, studies have shown that positively charged AMPs interact with negatively charged microbial cell walls (Zasloff, 2002; Jenssen et al., 2006). The mechanism underlying this interaction may be membrane disruption and the formation of a carpet, a barrel or a membrane clump, or internalization causing dysfunction of the intracellular target (Powers and Hancock, 2003; Giuliani et al., 2007).

Antimicrobial peptides have been identified in the venom of arthropods, such as wasps, scorpions, and bees. The AMPs derived from scorpion venom can be divided into two groups according to the presence of a disulfide bridge (DB group) or the absence of a disulfide bridge [non-disulfide bridge (NDB) group]. Small peptides from scorpion venom belonging to the NDB group were characterized as being fungicidal and bactericidal (Dai et al., 2001, 2002; Zeng et al., 2004, 2013; Gao et al., 2009). For example, ToAP2 from *Tityus obscurus* displayed a low MIC and was able to destroy a *Candida albicans* biofilm (Guilhelmelli et al., 2016). The mature peptide NDBP-5.5 from *Hadrurus gertschi* venom (Schwartz et al., 2007) possesses 13 amino acids and also belongs to the NDB group of peptides, being classified as NDBP-5.5, and therefore may exert microbicidal activity.

The *Mycobacterium abscessus* complex is composed of rapid-growing mycobacteria that cause human and animal infections. Such infections predominantly affect the skin, lungs, and connective tissue (Griffith, 2007; Cardoso et al., 2008; Kim et al., 2008; Duarte et al., 2009). The incidence of *M. abscessus* infections has been increasing and becoming more widespread over recent years (Lee et al., 2015). The *M. abscessus* complex comprises three subspecies: *M. abscessus* subsp. *abscessus, M. abscessus* subsp. *massiliense*, and *M. abscessus* subsp. *bolletii* (Adékambi et al., 2004; Lee et al., 2015). Several cases of infection with *M. abscessus* subsp. *massiliense* were reported in Brazil in 63 different hospitals. The bacteria recovered from theses different outbreaks presented resistance to glutaraldehyde as well as to antimicrobial drugs (Duarte et al., 2009). Additionally, this subspecies of *M. abscessus* was shown to be more virulent and pathogenic than the other species of the group (Sousa et al., 2010; Shang et al., 2011). The treatment of infections caused by these bacilli depends on the species, but involves some combination of clarithromycin (CLR), amikacin (AMK), cefoxitin (FOX), and/or imipenem (IPM) (Cardoso et al., 2011; Tettelin et al., 2014). However, infections caused by drug-resistant bacilli have emerged in recent years (Kim et al., 2008; Choi et al., 2012). Thus, it is important to search for new drugs or molecules that could substitute, or be combined with, existing treatments.

The aim of this study was to determine the activity, both *in vitro* and *in vivo*, of NDBP-5.5 against *M. abscessus* subsp. *massiliense*.

## Materials and Methods

### NDBP-5.5

Peptide NDBP-5.5 (UniProt P0C8W1) was identified as previously described (Schwartz et al., 2007) and was synthesized by C-terminal amidation using FastBio LTDA (Ribeirão Preto, SP, Brazil). NDBP-5.5 presented >95% of purity. The molecular mass and sequence of the synthetic NDBP5.5 were confirmed by MALDI-TOF/TOF MS (UltraFlex III, BrukerDaltonics, Germany) and LIFTTM (MS/MS) as previously described (Guilhelmelli et al., 2016).

### Bioinformatic Sequence Similarity Analysis

Multiple sequence alignment of the obtained sequences of peptide NDBP-5.5 was performed using ClustalW2 and BioEdit software (Larkin et al., 2007). Structural predictions, as shown in an alpha helix diagram, were generated by the HeliQuest server^1^.

### Peptide Stability in Aqueous Solution

Molecular dynamics (MD) simulations were performed with Gromacs 5.1.3 (Berendsen et al., 1995; Yu, 2012) using force field AMBER99SB-ILDN (Yildirim et al., 2010) in order to explore the stability (or instability) of the peptide in water. Initially, the peptide was solvated with a box cubic wall distance of 10 Å using water model TIP3P (Mahoney and Jorgensen, 2000). The system was neutralized by adding the required number of counter ions. The particle mesh Ewald method (Darden et al., 1993) was used to evaluate electrostatic interactions with periodic boundary conditions considered in all directions from box. The system was initially subjected to minimization using the steepest descent energy. The simulations were completed when the tolerance of 1000 kJ/mol was no longer exceeded. The next four stages consisted of a series of equilibration simulations to slowly relax the system without deviating from the initial conformation. The first two stages consisted of 50 ps MD simulations in NVT and NPT ensembled at 300 K with a restraint of 50 kcal/mol/Å on the peptide atoms. In the next stage, the simulations were performed during 0.5 ns without restraint in NPT ensembled at 300 K. Finally, the simulations were performed for 100 ns with a constant temperature of 300 K, 1 atm pressure, time-setup of 2 fs, and without any restriction of peptide conformations. All information concerning the trajectory of these times were collected every 50 ps. The equilibration of the trajectory was evaluated by monitoring the equilibration of quantities, such as the root-mean-square deviation (RMSD) (Coutsias et al., 2004) of the non-hydrogen atoms, kinetic energy, total energy, and potential energy.

The program g_cluster (GROMACS tool) was used to determine the conformations that best represented the peptide structures of the entire trajectory. The algorithm gromos, as described by Daura et al. (1999), was selected for this purpose. A cutoff = 0.2 nm for the clusters was used considering the profile of the RMSD. The clusters were determined using the non-hydrogen atom RMSD values.

### *Mycobacterium abscessus* subsp. *massiliense* Cultures

The mycobactericidal activity of NDBP-5.5 was determined *in vitro* using three clinical isolates randomly selected from our laboratory collection: *M. abscessus* subsp. *massiliense* GO01, GO06, and GO08 (Cardoso et al., 2008) and the reference strain CRM0020 (Duarte et al., 2009). The bacteria were grown in Mueller–Hinton medium (MH, HIMEDIA) for 3 days at 35°C. Then, the cultures were adjusted to 1.5 × 10^8^ CFU/mL (0.5 McFarland scale) to determine the minimal bactericidal concentration (MBC).

*Mycobacterium abscessus* subsp. *massiliense* GO06 was used for the *in vivo* experiments (mice intravenous infection) (Sousa et al., 2010; das Neves et al., 2016). The bacterial suspension was diluted in PBS 0.05% Tween 80 and adjusted to 1 × 10^6^ CFU/mL.

### Animals

Macrophages were obtained from the bone marrow of BALB/c mice. IFN-γ KO mice were used to test the effect of peptide NDBP-5.5 on the treatment of *M. abscessus* subsp. *massiliense* infection. Female mice, of 7–9 weeks-of-age, were obtained from the animal facilities at the Institute of Tropical Pathology and Public Health at the Federal University of Goiás (IPTSP/UFG). All animal handling procedures and protocols were performed according to the National Counsel for animal experimentation recommendations (Conselho Nacional de Controle de Experimentação Animal -CONCEA). The Animal Ethics Committee at the Federal University of Goias approved the protocols (Comitê de Ética no uso de animais da Universidade Federal de Goiás (No: 016/14).

### Minimal Bactericidal Concentration (MBC) of NDBP-5.5

The MBC determination was adapted from ATS/IDSA guidelines (Griffith et al., 2007). The lyophilized peptide NDBP-5.5 was diluted in 3% DMSO. *M. abscessus* subsp. *massiliense* cultures, grown as described previously, were adjusted to 100 CFU/100 μL and added to 96-well plates. The peptide solution was serially diluted in PBS (13–400 μM), and 100 μL of each dilution was added to the wells. Each NDBP-5.5 peptide dilution was tested in triplicate. The plates were incubated for 3 days at 35°C. All test plates contained control wells of medium only, *M. abscessus* subsp. *massiliense* isolates as positive controls, or *M. abscessus* subsp. *massiliense* isolates treated with CLR (1.34 μM) as a negative control. After 3 days of culture, the wells were homogenized and transferred to an MH agar plate to calculate the number of CFU per treatment. The MBC was determined and the percentage of inhibition in relation to bacterial growth in the medium without treatment was calculated.

### Hemolysis Assay

The toxicity of NDBP-5.5 against human red blood cells was evaluated as described previously (Ramírez-Carreto et al., 2012). Ten milliliters of peripheral blood from a healthy subject (without any symptoms of disease) were collected. The protocol was approved by the Ethics Committee of the Federal University of Goias (protocol number: 055/2009) and the participant signed a consent form. Briefly, red blood cells were obtained by centrifugation of the blood at 1000 × *g*. The cells were then washed with PBS, adjusted to 1 × 10^8^ cells/mL, and 100 μL were added to the wells of a 96-well plate. The NDBP-5.5 solution was diluted to 8, 4, 2, and 1× the peptide MBC value (200 μM) and 100 μL was added to the wells. The plates were incubated for 1 h at 37°C, and after this period they were centrifuged (1000 × *g*) and the absorbance was determined at 540 nm. Red blood cells treated with Triton-100X (0.1%) were used as positive controls and red blood cells only were used as negative controls. The assay was performed in triplicate and repeated twice. The percentage of hemolysis was obtained using the formula: % of red cell lysis = 100× [(test-PBS)/(Triton-PBS)].

The therapeutic index (TI) was determined according to Wang et al. (2009) by dividing the concentration of NDBP-5.5 that caused 10% hemolysis by the MBC value.

### Mycobactericidal Activity of NDBP-5.5 in Bone Marrow-Derived Macrophages Infected with *M. abscessus* subsp. *massiliense*

BALB/c mice (*n* = 4) were euthanized and the femur bone marrow was extracted, homogenized and plated with 10 ng/mL of GM-CSF as described previously (Becker et al., 2012). After 3 days of culture, the medium was supplemented with 10 ng/mL of GM-CSF. After 7–10 days of culture, the macrophages were transferred to a 96-well plate at 10^5^ cells/well. After a 1 h incubation at 37°C in a CO_2_ incubator, 10^6^ CFU/well of *M. abscessus* subsp. *massiliense* were added to the cultures [multiplicity of infection (MOI), 10:1] and further incubated for 3 h. Then, the cell cultures were washed with RPMI-1640 (HIMEDIA), containing 10% fetal bovine serum, 1 mM sodium pyruvate, 100 U/mL penicillin, 100 g/mL streptomycin, 2 mM L-glutamine, and 2 mM non-essential amino acids.

After this procedure, the infected macrophages were treated with 200 μM of NDBP-5.5 (MBC = 200 μM) prepared in RPMI-1640 media. After 72 h, the cells were treated with 200 μL of deionized water, and after homogenization, the suspensions were plated on MH agar plates for culturing to determine the number of CFU. Controls containing only infected macrophages or macrophages treated with CLR were added to all of the 96-well test plates.

### *In vivo* Mycobactericidal Activity of NDBP-5.5

IFN-γ KO mice were intravenously infected with 10^6^ CFU of *M. abscessus* subsp. *massiliense*. After 18 days of infection, the mice were treated with NDBP-5.5 (2 mg/kg, *n* = 12 animals; or 1 mg/kg, *n* = 12 animals) or CLR (200 mg/kg, *n* = 12 animals) over 8 consecutive days. An infection group treated with PBS (*n* = 12) was used as a control. The animals were treated by intraperitoneal injection with a volume of 100 μL as previously described (das Neves et al., 2016).

To monitor the infection with *M. abscessus* subsp. *massiliense*, the day following infection and 18 days post-infection, three animals per time and group were euthanized, and the lungs, spleens, and livers were collected to determine the bacillary count.

Twenty-seven-days post infection (1 day after the end of treatment), the animals were euthanized (*n* = 6), and spleens, livers, and lungs were collected to determine the bacillary load.

To establish the bacterial load, the organs were homogenized in PBS 0.05% Tween 80, serially diluted, and plated in MH agar. The percentage reduction in bacterial numbers was calculated using the number of CFU obtained from the organs of animals infected with *M. abscessus* subsp. *massiliense* without treatment and the number of CFU from the groups treated with NDBP-5.5 or CLR.

### Histology

After 28 days of infection, mice were euthanized and the organs were collected. The right lung caudal lobe from each animal was fixed in 10% paraformaldehyde and blocked in paraffin. The tissues were sectioned (at 5-μm), stained with hematoxylin and eosin, and observed under an optical microscope (Axio scope A1; Carl Zeiss, Oberkochen, Germany).

### Statistical Analysis

The data were tabulated using Excel software and the average and standard deviation values were calculated. To evaluate statistical differences among groups, the software GraphPad Prism 6.0 was used. The variances among groups were evaluated by one-way ANOVA, followed by the Tukey test. Differences were considered statistically significant when *p* < 0.05.

## Results

### Peptide Characteristics

**FIGURE 1:**
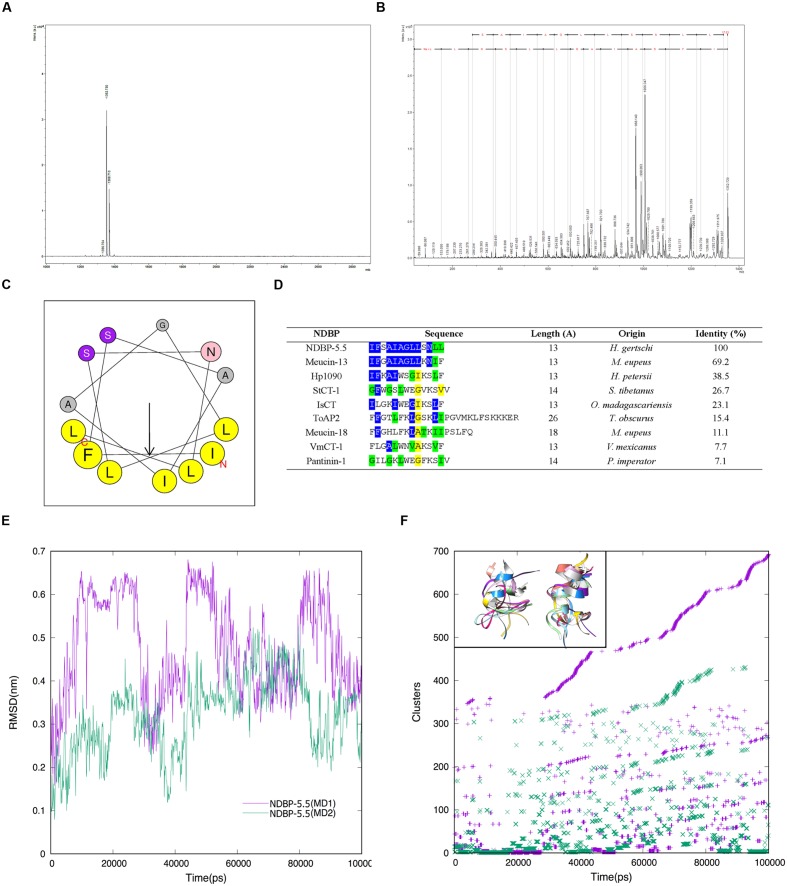
**Spectrophotometry and bioinformatic analysis of the NDBP-5.5.** **(A)** Mass spectrometry of the synthesized NDBP-5.5 peptide obtained by MALDI TOF/TOF. **(B)** Mass fragmentation pattern (MS/MS) obtained from the monoisotopic mass [M+Na]+ = 1532.73 Da ion. **(C)** Helicoidal wheel diagram (yellow residues: non-polar and hydrophobic; purple residues: polar; gray residues: neutral; pink residue: asparagine). C, carboxyl terminal end; N, amino terminal end. **(D)** Multiple alignment of the sequences of NDBP-5.5, IsCT, Meucin-13, Meucin-18, VmCT-1, StCT-1, Hp1090, and Pantinin-1. Blue, identical amino acids; green, highly homologous amino acids to those in the NDPB-5.5 sequence; yellow, higher homology between the peptides. **(E)** RMSD dynamic profiles obtained for the MD1 (purple) and MD2 (green) simulations of NDBP-5.5 peptide over 100 ns. **(F)** Cluster analysis of the trajectory obtained for a cutoff of 0.2 nm in order to select the main structures during the simulations. The clusters were determined using the non-hydrogen-atom RMSD values. Inserted figures shown at the top left represent 10 cluster structures from MD1 (left) and MD2 (right) independent simulations.

A previously described cDNA sequence of a peptide derived from *H. gerstchi* venom (Schwartz et al., 2007) and comprising 13 amino acids (IFSAIAGLLSNLL) with C-terminal amidation, namely NDBP-5.5, was synthetized and the sequence was confirmed by MALDI-TOF/TOF (**Figures 1A,B**). The predicted secondary structure^1^ revealed an alpha helix with amphipathic characteristics containing a hydrophilic region (A,S,S,G,N,A) and a hydrophobic region (L,F,L,I,L,I,L) (**Figure 1C**). Sequence alignment of NDBP-5.5 with other scorpion venom peptides from the same family of peptides (from the NDB group) showed highest similarity (69.2%) with Meucin-13 from *Mesobuthus eupeus* venom (**Figure 1D**). MD simulations from the PEP-FOLD model were performed in order to verify the stability of the NDBP-5.5 in water under two independent trajectories (different initial conditions). **Figures 1E,F** shows the RMSD evolution from initial model (PEP-FOLD structure) for MD1 and MD2 simulations. For both simulations, the peptide was not able to remain stable around a particular conformation, even to any conformation other than the helical structure. It can be verified from cluster analysis of the trajectory, that most of the structures were unstable for any time during the simulation (**Figure 1F**).

### NDBP-5.5 Antimicrobial Activity and Hemolysis Induction

**FIGURE 2:**
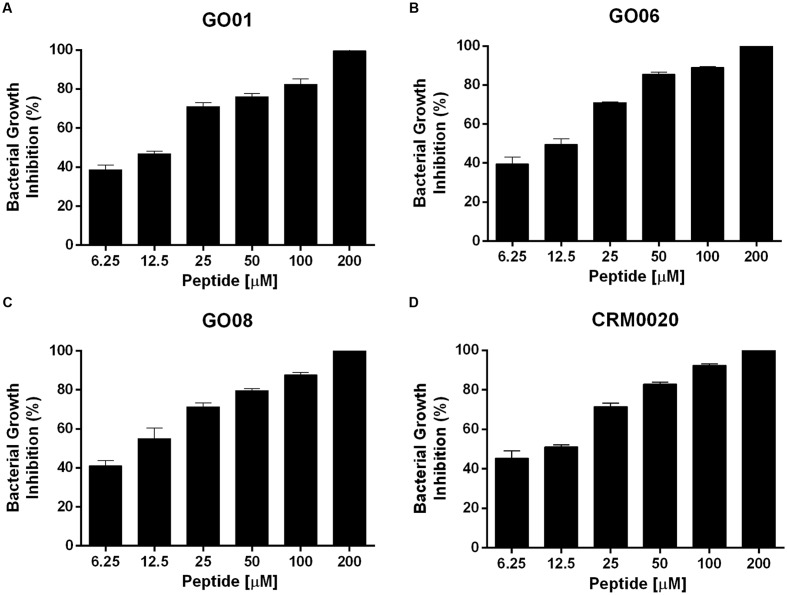
**Minimal bactericidal concentration (MBC) of NDBP-5.5 against *Mycobacterium abscessus* subsp. *massiliense*.** Three different *M. abscessus* subsp. *massiliense* clinical isolates and the reference strain CRM0020 were cultivated with different concentrations of NDBP-5.5 for 24 h. The percentage of growth inhibition was calculated by plating serial dilutions of each culture and determining the number of colony-forming units (CFUs). **(A)** Isolate GO01; **(B)** isolate GO06; **(C)** isolate GO08; **(D)** reference strain CRM0020. The MBC was determined as the lowest concentration that completely inhibited mycobacterial growth. These results are the mean values of three independent experiments.

NDBP-5.5 inhibited the growth of all *M. abscessus* subsp. *massiliense* isolates evaluated (**Figure 2**). Using a range of clinical isolates and a reference strain (CRM0020), the MBC of NDBP-5.5 was determined to be 200 μM.

**FIGURE 3:**
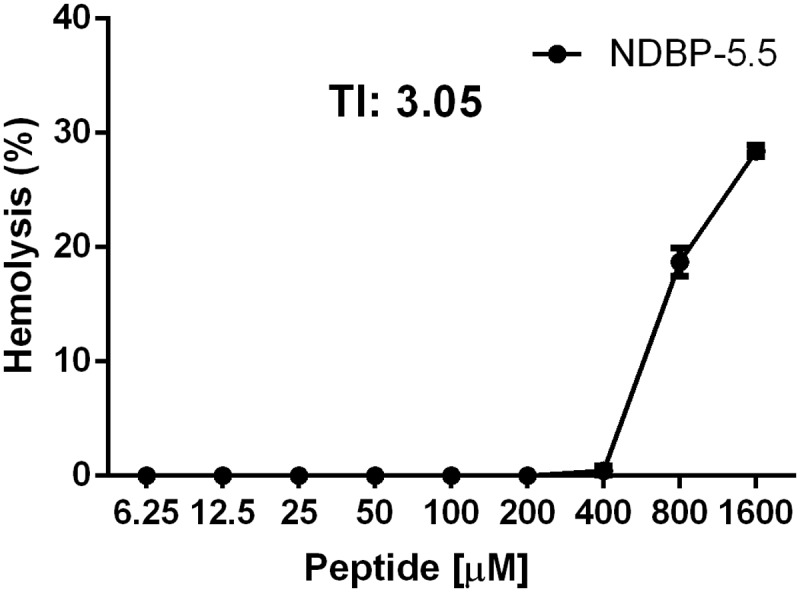
**Hemolytic activity of NDBP-5.5.** Human red blood cells were incubated with the NDBP-5.5 in concentrations ranging from 6.25 to 1600 μM. The therapeutic index (TI) calculated based on the percentage of hemolytic activity at the MBC is shown.

As shown in **Figure 3**, NDBP-5.5 displayed low hemolytic activity. When the peptide was used at a concentration eight-times higher (1600 μM) than that of the MIC, 39% hemolysis occurred (**Figure 3**). The concentration of NDBP-5.5 that caused 10% hemolysis was calculated (611.8 μM) and, as suggested by Wang et al. (2009), used to estimate the TI, by dividing by the MBC value (200 μM). The TI was determined as 3.05.

### Antimicrobial Activity of NDBP-5.5 against *M. abscessus* subsp. *massiliense* in Infected Macrophages

**FIGURE 4:**
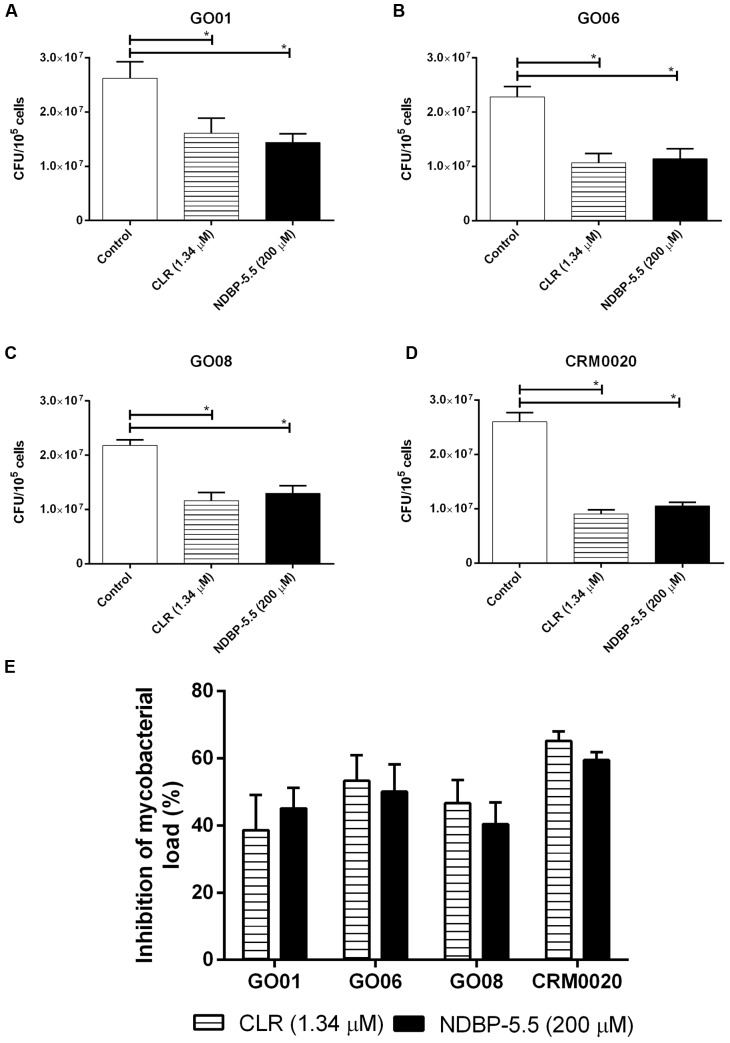
**Effect of NDBP-5.5 treatment of macrophages infected with different strains of *M. abscessus* subsp. *massiliense*.** Bacillary load after treatment of macrophages infected with **(A)** isolate GO01, **(B)** isolate GO06, **(C)** isolate GO08, **(D)** CRM0020, and respective controls. **(E)** Shows the percentage of bacillary load reduction among all strains treated with NDBP-5.5 or CLR. These results are the mean values of three independent experiments. ^∗^*p* < 0.05 statistically significant difference between the test groups and the infected only group.

Since NDBP-5.5 presented low toxicity, we decided to test its function against phagocytosed bacteria. The treatment of infected macrophages (MOI, 1:10) with NDBP-5.5 or CLR reduced the bacterial load at similar levels (**Figure 4**). The highest bacterial load reduction was observed with the reference strain (60% reduction; **Figure 4E**).

### NDBP-5.5 Treatment of IFN-γ KO Mice Infected with *M. abscessus* subsp. *massiliense*

**FIGURE 5:**
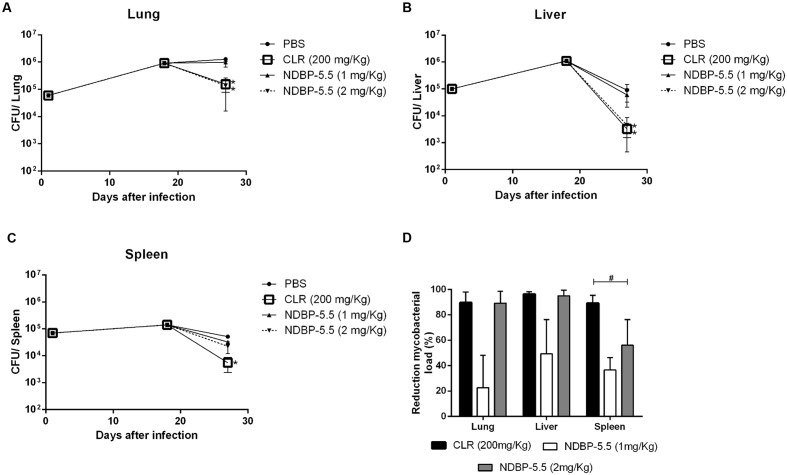
**Bacillary load reduction in IFN-γ KO mice infected with *M. abscessus* subsp. *massiliense*.** **(A)** Lung bacillary load; **(B)** spleen bacillary load; **(C**) liver bacillary load. **(D)** Percentage of bacillary load reduction in the three different organs. These results are the mean values of three independent experiments. ^∗^*p* < 0.05 statistically significant difference between the test groups and the only infected group and the PBS treatment group. ^#^*p* < 0.05 statistically significant difference between the CLR and NDBP-5.5 treated groups.

**FIGURE 6:**
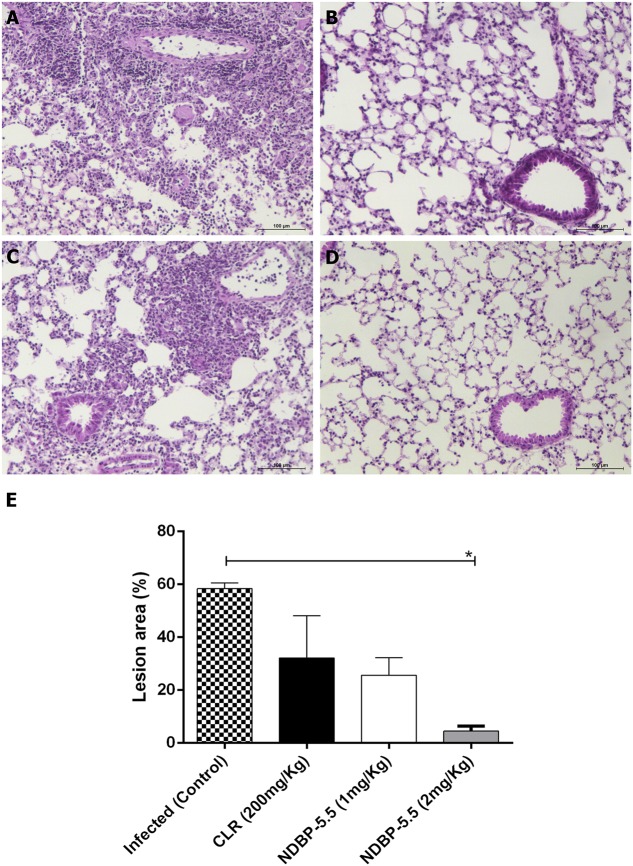
**Lung inflammation in mice infected with *M. abscessus* subsp. *massiliense* and treated with NDBP-5.5 (1 or 2 mg/kg) or CLR.** After 8 days of treatment, the animals were euthanized and their lungs were processed and stained with hematoxylin and eosin. **(A)** Infected control group: diffuse lung infiltration of mononuclear cells and increase of arteriolar wall thickness. **(B)** Group infected and treated with CLR: periarteriolar mononuclear cells cuffs; alveolar septum thickness. **(C)** Group infected and treated with NDBP-5.5 (1 mg/kg): diffuse lung infiltration of mononuclear cells and increase of arteriolar wall thickness. **(D)** Group infected and treated with NDBP-5.5 (2 mg/kg): discrete mononuclear cells infiltration. **(E)** Histology score of lesion areas. Score obtained by AxioVision 4.9.1 software, through the ratio between lesioned area and the whole image. The data were evaluated in the whole lung. Values showed in percentage (%). ^∗^*p* < 0.05 statistically significant difference between the test groups and the group only infected.

Mice susceptible to *M. abscessus* subsp. *massiliense* infection were used to test the *in vivo* activity of NDBP-5.5. Using 2 mg/kg of NDBP-5.5, the bacterial load was significantly reduced in the lungs and liver. The bacterial load reduction observed at this dosage (2 mg/kg) was similar to that observed for CLR (**Figures 5A–C**). Evaluating the lung inflammatory lesions of the infected animals, it was possible to observe diffuse inflammatory lesions at 28-days-post-infection (**Figure 6A**), that were reduced when the animals were treated with CLR (**Figure 6B**). Treatment with NDBP-5.5 at 1 mg/kg resulted in inflammatory lesions that were similar to the lesions observed in the lungs of infected animals that were not treated with NDBP-5.5 (**Figure 6C**). The lung lesions induced by infection in IFN-γ KO mice were drastically reduced when the animals were treated with NDBP-5.5 at 2 mg/kg (**Figure 6D**). The score of the lesions were evaluated and showed that mice treated with NDBP-5.5 (2 mg/Kg) had reduced inflammatory lesions when compared to infected and not treated animals (**Figure 6E**).

## Discussion

Our findings demonstrate, for the first time, the antimicrobial activity of a peptide from scorpion venom against rapid-growing mycobacteria. NDBP-5.5 is an amphipathic peptide from the family of NDB peptides derived from the venom of *H. gertschi* that showed activity against *M. abscessus* subsp. *massiliense*, a rapid-growing *Mycobacterium* that has been associated with healthcare infections. NDBP-5.5 showed a MBC of 200 μM and, at the concentration tested, was not hemolytic. NDBP-5.5 reduced the bacterial load of infected macrophages, as well as infected mice, to similar levels as CLR, one of the antibiotics used to treat infections with these bacteria.

*In silico* sequence analysis of NDBP-5.5 revealed polar and hydrophobic regions suggesting amphipathic properties (Wimley, 2010). The high similarity observed between NDBP-5.5 and Meucin-13 peptide from *M. eupeus* venom, which has potent activity against Gram-positive bacteria (Gao et al., 2009), suggested that NDBP-5.5 may also exert bactericidal properties as both peptides share the same conserved hydrophobic amino acids.

NDBP-5.5 presented an MBC of 200 μM (266 μg/mL) against *M. abscessus* subsp. *massiliense*. This MBC value was high compared with that of other similar peptides, such as Polydim-1 derived from wasp (*Polybia dimorpha*) venom that presented an MBC of 60.8 μg/mL (12.5 μM) against the same species of mycobacteria (das Neves et al., 2016). The MBC was also high compared with other scorpion venom-derived peptides tested against other microorganisms (1–200 μM) (Dai et al., 2001; Gao et al., 2009; Yan et al., 2011; Ramírez-Carreto et al., 2012, 2015; Zeng et al., 2013); however, these peptides were not evaluated for their activity against mycobacteria. The ToAP2 from *T. obscurus*, which belongs to the same NDB family of peptides, presented a varied MBC of 3.12 to 200 μM when tested against *Candida* spp. (Guilhelmelli et al., 2016). Despite the high concentration needed for mycobactericidal action, NDBP-5.5 showed low toxicity even at concentrations eight-times higher than the MBC, corresponding to a TI of 3.05. By contrast, peptide Meucin-13 presented high toxicity at its MIC concentration (MIC = 6.5 μM) against bacteria and fungi (Gao et al., 2009). TIs varying from 0.5 to 12.1 have been found for AMPs tested against *Mycobacterium tuberculosis* (Ramírez-Carreto et al., 2015), nonetheless none of those peptides were tested in animals to confirm their ability to treat infection. Thus, NDBP-5.5 could be a promising drug candidate because it exerts mycobactericidal activity accompanied by low toxicity *in vivo*.

Meucin-13, which shows high similarity to NDBP-5.5, induces membrane modifications that culminate in the lysis of *Bacillus megaterium* cells (Gao et al., 2009). MD analysis revealed the instability of NDBP-5.5 in aqueous solution, suggesting its necessity of a non-polar environment such as membrane for its stabilization, and hence its possible target of action. Although not shown, MEV analysis of *M. abscessus* subsp. *massiliense* treatment with NDBP-5.5 induced agglomeration of the bacilli but did not appear to alter the mycobacterial envelope structure. It is possible that NDBP-5.5 acts by modifying the cell wall, without forming pores or lesion, but instead by parallel lining of the cell wall (carpet) causing perturbation of the osmotic mechanisms and consequently allowing leakage of intracellular content (Powers and Hancock, 2003; Nguyen et al., 2011), but this hypothesis needs further testing.

There is a direct relationship between polar charges in the hydrophilic region of a peptide and its hemolytic action (Chen et al., 2007). The presence of two polar residues in the hydrophilic region of NDBP-5.5 may be responsible for the low rate of cytotoxicity observed, due to reduced contact with the erythrocyte membrane. Accordingly, the Pantinin-3 AMP presented three polar residues, and was more toxic to erythrocytes than Pantinin-2 AMP that possessed only two polar residues in the same hydrophilic region (Zeng et al., 2013). Similarly, VmCT2 displayed three polar residues in its hydrophilic region and was shown to be more hemolytic in higher concentrations than VmCT1 with only one polar residue in the same region (Ramírez-Carreto et al., 2012).

Our experiments in infected macrophages revealed that the activity of NDBP-5.5 was not only *in vitro*, as a reduction of approximately 50% of the bacterial load was observed in infected macrophages, similar to the effects seen with CLR. This microbicidal action indicated the potential application of NDBP-5.5 in the treatment of infected animals. IFN-γ KO mice that are more susceptible to mycobacterial infections were used to test this hypothesis (Rottman et al., 2007; Shang et al., 2011). NDBP-5.5 treatment significantly reduced the bacillary load in the lungs and liver of *M. abscessus* subsp. *massiliense*-infected IFN-γ KO mice in a similar manner as other AMPs against *Mycobacterium tuberculosis* (Rivas-Santiago et al., 2013).

In this study, it was not possible to determine the mechanism of microbicidal induction by NDBP-5.5. The specific activity of this AMP on mycobacteria and on macrophages may be mediated via different targets and different modes of action.

The administration of a combination of antimicrobials can reduce the propensity for developing resistance and is a promising strategy to treat diseases caused by drug-resistant bacteria (Huang et al., 2010). NDBP-5.5 could therefore be combined with conventional antimicrobials to treat rapid-growing mycobacterial infections. The treatment of this group of mycobacteria requires more than one drug to effectively clear the pathogen. To test whether NDBP-5.5 would have an addictive or synergic effect with CLR, *M. abscessus* subsp. *massiliense* was exposed to different drug concentration combinations. *In vitro*, no association between the tested drugs was observed (data not shown). Thus, it seems that the major advantage supported by the data presented here is that the peptide might reduce the extensive inflammatory reactions induced by the infection nonetheless, bacterial clearance will need the aid of others antibiotics.

In summary, the AMP NDBP-5.5 derived from the *H. gertschi* scorpion displayed antibacterial activity against *M. abscessus* subsp. *massiliense*, both *in vitro* and *in vivo*, along with low hemolytic activity. Further studies are now needed to understand the mechanism of action of this peptide before it can potentially be used as an auxiliary treatment for rapid-growing mycobacteria infections.

## Ethics Statement

This study was carried out in accordance with the recommendations of Conselho Nacional de Controle de Experimentação Animal -CONCEA. The protocol was approved by the Comitê de Ética no uso de animais da Universidade Federal de Goiás (No: 016/14).

## Author Contributions

MT, RdN, and BdPOS carried out the experiments and the NDBP-5.5 mycobactericidal characterization. MM and ABdS carried out the MS experiment. BdPOS contributed to the bioinformatics results. RD performed the molecular dynamics of the peptide. ES selected the AMP to be used in this work. AJ-K and AK designed the experiments and supervised all work. MT, RdN, BdPOS, and AJ-K wrote the manuscript. All authors revised the manuscript and approved the final version.

## Conflict of Interest Statement

The authors declare that the research was conducted in the absence of any commercial or financial relationships that could be construed as a potential conflict of interest.

## Footnotes

1 http://heliquest.ipmc.cnrs.fr
